# The status of academic interventional radiologists in Germany with focus on gender disparity: how can we do better?

**DOI:** 10.1186/s42155-024-00456-4

**Published:** 2024-05-16

**Authors:** Sophia Freya Ulrike Blum, Cornelia Lieselotte Angelika Dewald, Lena Becker, Emona Staudacher, Mareike Franke, Marcus Katoh, Ralf-Thorsten Hoffmann, Stefan Rohde, Philip Marius Paprottka, Frank Wacker, Kerstin Westphalen, Philipp Bruners, Bernhard Gebauer, Marco Das, Wibke Uller

**Affiliations:** 1 Institute and Polyclinic for Diagnostic and Interventional Radiology, University Hospital Carl Gustav Carus, Technical University Dresden, Fetscherstraße 47, D-01307 Dresden, Germany; 2 Institute for Diagnostic and Interventional Radiology, Hannover Medical School, Carl- Neuberg-Straße 1, 30625 Hannover, Germany; 3 Department of Diagnostic and Interventional Radiology, RWTH Aachen University, Pauwelsstraße 30, 52074 Aachen, Germany; 4 Radiology and Nuclear Medicine, Canton Hospital Lucerne, Lucerne, Switzerland; 5 Department for Diagnostic and Interventional Radiology, HELIOS Hospital Krefeld, Lutherplatz 40, 47805 Krefeld, Germany; 6 Department of Radiology and Neuroradiology, Klinikum Dortmund gGmbH, Beurhausstraße 40, 44137 Dortmund, Germany; 7 Department of Interventional Radiology, Klinikum rechts der Isar, Technical University Munich, Ismaninger Straße 22, 81675 München, Germany; 8 Department of Radiology, DRK Hospital Berlin, Salvador-Allende-Straße 2-8, 12559 Berlin, Germany; 9 Department of Radiology, Charité University Medicine Berlin, Südstraße 3, 13353 Berlin, Germany; 10 Department of Radiology, Helios Hospital Duisburg, Dieselstraße 185, 47166 Duisburg, Germany; 11 Department of Diagnostic and Interventional Radiology, Faculty of Medicine, Medical Center University of Freiburg, University of Freiburg, Hugstetter Straße 55, 79106 Freiburg im Breisgau, Germany

**Keywords:** Interventional radiology, IR research, Gender disparity, Academic IR

## Abstract

**Purpose:**

The aim was to characterize the framework conditions in academic interventional radiology (IR) in Germany with focus on differences between genders.

**Materials and methods:**

After IRB approval, all members of The German Society for Interventional Radiology and Minimally Invasive Therapy (*n* = 1,632) were invited to an online survey on work and research. Statistical comparisons were undertaken with the Fisher’s exact test, Wilcoxon rank sum test or Pearson’s Chi-squared test.

**Results:**

From 267 available questionnaires (general response rate 16.4%), 200 were fully completed. 40% of these (78/200) were involved in research (71% men vs. 29% women, *p* < 0.01) and eligible for further analysis. Of these, 6% worked part-time (2% vs. 17%, *p* < 0.05). 90% of the respondents spent less than 25% of their research during their paid working hours, and 41% performed more than 75% of their research during. leisure time. 28% received exemption for research. 88% were (rather) satisfied with their career. One in two participants successfully applied for funding, with higher success rates among male applicants (90% vs. 75%) and respondents with protected research time (93% vs. 80%). Compared to men, women rated their entrance in research as harder (*p* < 0.05), their research career as more important (*p* < 0.05), felt less noticed at congresses (93% vs. 53%, *p* < 0.01), less confident (98% vs. 71%, *p* < 0.01), and not well connected (77% vs. 36%, *p* < 0.01).

**Conclusion:**

Women and men did research under the same circumstances; however, women were underrepresented. Future programs should generally focus on protected research time and gather female mentors to advance academic IR in Germany.

**Supplementary Information:**

The online version contains supplementary material available at 10.1186/s42155-024-00456-4.

## Background

Interventional radiology (IR) is an integral part of modern health care provision [1, 2]. While in Europe and North America half or even more of the students are women, IR has the largest disparity of any radiological subspecialty [3, 4], and some countries even report a shortage of interventional radiologists [5]. In 1998, the gender ratio among German medical students was balanced. In the meantime, the proportion of women has risen to 64% [6]. Considering the relatively high proportion of women in other specialties such as gynecology, child and adolescent psychiatry, dermatology, and pediatrics, there is concern that the additional female graduates in Germany will choose not to become radiologists or IR physicians [7]. Given the continual advancement of innovative techniques and expanding indications in IR, it should be of great interest to encourage and foster interest in IR research in the future and to facilitate the participation of women in pursuing research careers within this captivating and diverse field. Unlike the US system, e.g., interventional and diagnostic radiology are not sharply separated in training. There is no separate training pathway for IR, as IR is not a subspecialization. Another difference to the US is that there is generally no distinction between purely clinical and purely academic fellowships. Research activities are centered around university hospitals. Research is carried out alongside clinical work unless the researcher has (protected) research time. This depends either on the exemption granted by the chief physician or on the raising of funds that will finance the exemption. Two-thirds of the institutions offer a dedicated IR rotation, which usually occurs at the end of the professional training and lasts only six months [8]. Many young physicians try to utilize IR research as an early entrance into clinical IR, giving IR mentors a special responsibility for promoting young talents.

Academic IR is a vital requirement to further advance our diagnostic and therapeutic skills in patient care. In a recent statement paper, specific recommendations were provided on how to establish a culture of excellence in IR, with a particular emphasis on promoting academic engagement [9]. Despite the positive aspects, academic work generally encounters barriers such as competition for grants, long or unpaid working hours, administrative tasks, and full teaching schedules but also lack of role models/ effective mentoring and guidance as well as conflicts with family responsibilities [10]. All over the world, fewer female residents are involved in academic activities [11]. Consequently, women are still underrepresented in academic IR [12]. This underrepresentation manifests in the small number of female first and last authorships in IR compared to other radiological subspecialties [13]. According to a study of Bernard et al., the rate of female first and last authors in IR had a significant smaller increase than publications in other subspecialties. Remarkably, Germany, despite its high output of articles about IR even ranked among the countries with the lowest proportion of female first and last authorships in Europe [13]. While female IR researchers from other countries have been catching up in the last decades, no corresponding trend was recorded for German IR researchers [14, 15].

The aim of this study was to characterize the overall barriers and opportunities for academic IR in Germany with a special focus on how this adversely affects women.

## Materials and methods

Approval from the local ethics committee was obtained. Between November 2021 and February 2022, all members (1,632, 86% men [16]) of The German Society for Interventional Radiology and Minimally Invasive Therapy (DeGIR) were invited to participate in an anonymous and voluntary online survey on the situation of interventional radiologists, particularly those engaged in research activities. The academic subset of the survey specifically targeted networking, funding, working hours and time spent on research (Supplement 1). Demographical data was obtained from the main survey. Statistical analyses were performed with RStudio (2021.09.0). Descriptive statistics with respective percentages were used. For normally distributed data, standard deviations were given. For non-normally distributed data, median with interquartile range was displayed. To test for significant differences, Fisher’s exact test, Wilcoxon rank sum test or Pearson’s Chi-squared test were used. A p-value < 0.05 was considered statistically significant. To comply with data privacy protection and to obtain valid statistical results, very small groups were either aggregated (number of children) or omitted (gender identity) for analytic statistics.

## Results

Overall, 267 colleagues responded, with 200 fully evaluable questionnaires (gross response rate 16.4%). Of the 200 respondents (net response rate 12.2%), 121 (60.5%) indicated that they were men, 76 (38%) women, and 3 (1.5%) non-binary. This resulted in gender-specific response rates of 8.6% for men and 33.3% for women. Of the respondents who did not participate in IR research, 59 (52.7%) were men, 52 (46.4%) were women, and 1 (0.9%) was non-binary. Eighty respondents (40%) stated they participated in IR research and provided completed questionnaires. Of these, 23 respondents (28.7%) were women, and 2 (2.5%) were non-binary. Because of the underrepresentation of non-binary researchers and the obligation of data privacy, 78 questionnaires of researching women and men were included for further statistical analyses resulting in 39% of all respondents (78/200).

### Demographics

Demographic data are displayed in Table 1. Overall, two thirds of all respondents were younger than 46 years. A majority of the female academic interventional radiologists fell within this age group, whereas one third of the men were older than 45, leading to a significant heterogeneity among the groups (*p* = 0.017).

**Table 1:** Demographics of the respondents

	Overall, *N* = 78^1^	women, *N* = 23^1^	men, *N* = 55^1^	*p*-value
**age**				0.017^2^
< 30	5 (6.4%)	3 (13%)	2 (3.6%)	
31–45	44 (56%)	17 (74%)	27 (49%)	
46–60	23 (29%)	3 (13%)	20 (36%)	
> 60	6 (7.7%)	0 (0%)	6 (11%)	
**level of education**				0.034^2^
resident	12 (15%)	6 (26%)	6 (11%)	
specialist	8 (10%)	5 (22%)	3 (5.5%)	
senior physician	36 (46%)	8 (35%)	28 (51%)	
chief physician	22 (28%)	4 (17%)	18 (33%)	
**hospital type**				0.2^2^
other hospital	2 (2.6%)	0 (0%)	2 (3.6%)	
teaching hospital	54 (69%)	19 (83%)	35 (64%)	
university hospital	22 (28%)	4 (17%)	18 (33%)	
**number of beds**				0.9^2^
50–199	1 (1.3%)	0 (0%)	1 (1.8%)	
200–399	1 (1.3%)	0 (0%)	1 (1.8%)	
400–799	20 (26%)	5 (22%)	15 (27%)	
> 800	56 (72%)	18 (78%)	38 (69%)	
**income**				0.005^2^
equal	17 (22%)	6 (26%)	11 (20%)	
main provider	57 (73%)	13 (57%)	44 (80%)	
side provider	4 (5.1%)	4 (17%)	0 (0%)	
**employment status**				0.025^2^
full-time	73 (94%)	19 (83%)	54 (98%)	
part-time	5 (6.4%)	4 (17%)	1 (1.8%)	

^1^n (%)

^2^Fisher’s exact test

Overall, 28% of this collective were chief physicians (22/78) and 46% were senior physicians (36/78). Of the chief physicians 18% were women and of the senior physicians 22% were women. Furthermore, the proportion of women holding the position of chief physician, i.e. chief of general and interventional radiology, was only half as large compared to the men and more female residents and specialists had taken part in the survey (*p* = 0.034).

Regarding hospital type and number of beds, no differences in the distribution of men and women was found. Overall, most of the respondents worked full-time. Men were more often the main provider in the family and almost exclusively worked full-time (*p* = 0.005). The women working part-time belonged to the age group between 31 and 45 years.

### Research and working conditions

**Fig. 1:**
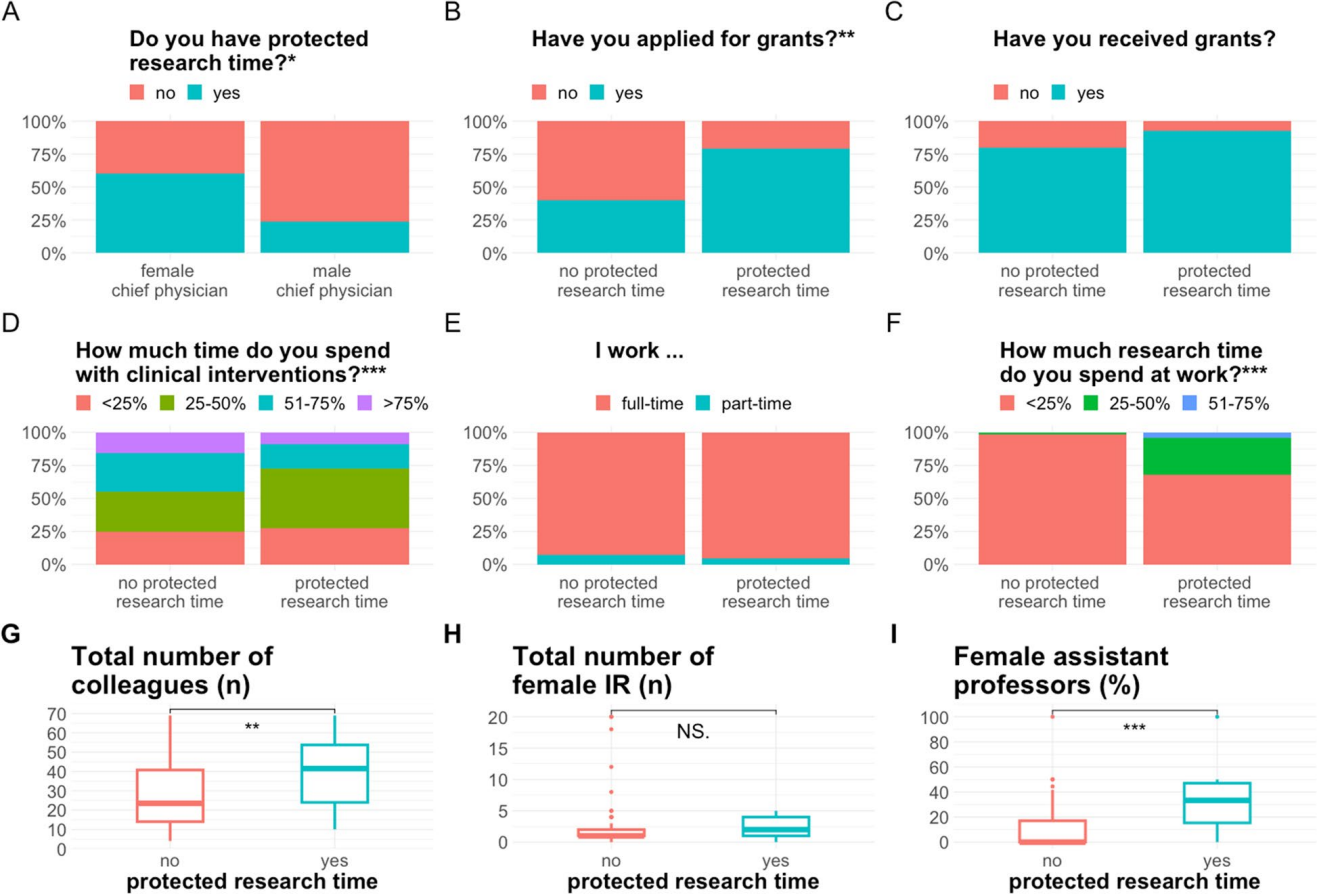
Bar graph and boxplots summarizing the questions about protected research time. (A) Gender of chief physician and rate of respondents who are given protected research time. (B) Protected research time and rate of respondents who applied for grants. (C) Protected research time and rate of respondents who received grants. (D) Protected research time and rate of clinical interventions as percentage of total working time as indicated by the respondents. (E) Protected research time and employment status of the respondents. (F) Protected research time and rate of research done at work as percentage of total working time as indicated by the respondents. Lower row with boxplots demonstrating protected research time given on the x-axes and number of colleagues as indicated by the respondents in the survey. F) Number of colleagues and protected research time. (G) Total numbers of female IR colleagues and protected research time. (H) Number of female assistant professors and protected research time. Statistical significance markers: NS.- not significant; * *p* < 0.05; ** *p* < 0.01; *** *p* < 0.001

Table 2 gives detailed insights in the answers about research and working conditions. 40% of the respondents conducted research in IR. Although there were no significant differences in the gender of chief physicians and supervisors between men and women, women more often had women as research group leaders compared to men, and they more often indicated to have female IR colleagues in their department. Both genders did the majority of their scientific activities after paid working hours, while only a small proportion had time for research during paid working hours. Research leave was possible for both genders. Respondents with female chief physicians had a significantly higher likelihood of receiving protected research time (Fig. 1A, *p* = 0.026). Significantly more colleagues had applied for grants when they had protected research time (Fig. 1B, *p* = 0.004). More respondents with protected research time than those without protected research time were successful with their application for a grant, however not statistically significant (Fig. 1C, *p* = 0.4). They spent the same amount of time with clinical interventions as respondents, who did not have protected research time (Fig. 1D, *p* = 0.6). The ratio of full- and part-time working respondents was equally distributed between respondents without and with protected research time (Fig. 1E, *p* > 0.9). Additionally, more research was conducted after regular working hours among those participants who did not have access to protected research time (Fig. 1F, *p* < 0.001). Respondents who had protected research time obviously worked in institutions with a higher number of radiologists, and by a higher rate of female assistant professors, respectively (Fig. 1G, *p* = 0.006; Fig. 1I, *p* < 0.001). A large stake of all the supervisors and chief radiologists were men. Women reported more often to have a woman as research group leader than men (Table 2, *p* = 0.01). Although not significant, female interventional radiologists were surrounded by more assistant professors and a larger number of IR colleagues.

**Table 2:** Summary of the questions about research and working conditions

	Overall, *N* = 78^1^	women, *N* = 23^1^	men, *N* = 55^1^	*p*-value
**What is the gender of your chief physician?**				0.5^2^
women	10 (13%)	4 (17%)	6 (11%)	
man	68 (87%)	19 (83%)	49 (89%)	
**What is the gender of your supervisor?**				0.7^2^
woman	13 (17%)	3 (13%)	10 (18%)	
man	65 (83%)	20 (87%)	45 (82%)	
**What is the gender of your research group leader?**				0.010^2^
non-binary	2 (2.6%)	0 (0%)	2 (3.6%)	
woman	10 (13%)	7 (30%)	3 (5.5%)	
man	66 (85%)	16 (70%)	50 (91%)	
**What is the number of your colleagues?**	28 (15, 45)	36 (24, 45)	23 (14, 46)	0.2^3^
**How many interventional radiologists work at your department?**	6.0 (5.0, 9.0)	6.0 (4.5, 10.0)	6.0 (5.0, 8.0)	> 0.9^3^
**How many female interventional radiologists work at your department?**	1.00 (1.00, 3.00)	3.00 (1.00, 4.00)	1.00 (1.00, 2.00)	0.003^3^
**How much of your paid working time do you spend with interventions?**				0.4^2^
< 25%	20 (26%)	9 (39%)	11 (20%)	
25–50%	27 (35%)	7 (30%)	20 (36%)	
51–75%	20 (26%)	4 (17%)	16 (29%)	
> 75%	11 (14%)	3 (13%)	8 (15%)	
**Do you get time to do research? (yes, %)**	22 (28%)	7 (30%)	15 (27%)	> 0.9^4^
**How much of your research do you perform during paid working hours?**				0.4^2^
< 25%	70 (90%)	20 (87%)	50 (91%)	
25–50%	7 (9.0%)	2 (8.7%)	5 (9.1%)	
51–75%	1 (1.3%)	1 (4.3%)	0 (0%)	
**How much research time do you spend in your free time?**				0.7^2^
< 25%	19 (24%)	5 (22%)	14 (25%)	
25–50%	18 (23%)	4 (17%)	14 (25%)	
51–75%	9 (12%)	4 (17%)	5 (9.1%)	
> 75%	32 (41%)	10 (43%)	22 (40%)	

^1^n (%); Median (IQR)

^2^Fisher’s exact test

^3^Wilcoxon rank sum test

^4^Pearson’s Chi-squared test

### Children and family friendly environment

Two thirds of the men have children, which is double the amount of the women (Table 3). Regarding age groups, 65% of the respondents with children were between 31 and 45 years old and 33% were between 46 and 60 years. Among the respondents who had children, 4.7% were residents, 9.3% were specialists, 58% were senior physicians and 28% were chief physicians. Women indicated an 8.6-fold more often that they were primarily responsible for the children. 12% of the respondents with children worked part-time (4 women and 1 man), but no one without children did. Although most respondents indicated working in a family friendly environment, including the possibility for parental leave and daycare, 84% of all respondents agreed that it is harder for women to manage children and career. When asked about the barriers, the most frequent topics were traditional role models with women facing more family obligations and more downtime, e.g. because of sick-leave for a child (Supplement 2).

**Table 3:** Summary of the questions about children and family friendly environment

	Overall, *N* = 78^1^	women, *N* = 23^1^	men, *N* = 55^1^	*p*-value
**family status**				0.056^2^
partnership	68 (87%)	17 (74%)	51 (93%)	
single	10 (13%)	6 (26%)	4 (7.3%)	
**Do you have children? (yes, %)**	43 (55%)	8 (35%)	35 (64%)	0.037^3^
**number of children**				0.4^2^
1	12 (28%)	4 (50%)	8 (23%)	
2	20 (47%)	4 (50%)	16 (46%)	
3	8 (19%)	0 (0%)	8 (23%)	
4 or more	3 (7%)	0 (0%)	3 (8.6%)	
**Who is responsible for the children?**				0.003^2^
equal	14 (33%)	5 (62%)	9 (26%)	
no	26 (60%)	1 (12%)	25 (71%)	
yes	3 (7.0%)	2 (25%)	1 (2.9%)	
**Do you have the possibility for daycare? (yes, %)**	30 (70%)	6 (75%)	24 (69%)	> 0.9^2^
**Are you a single parent? (yes, %)**	2 (4.7%)	0 (0%)	2 (5.7%)	> 0.9^2^
**For women it is harder to manage children and career.**				0.6^2^
consent	37 (61%)	14 (74%)	23 (55%)	
rather consent	8 (13%)	2 (11%)	6 (14%)	
partial consent	6 (9.8%)	2 (11%)	4 (9.5%)	
less consent	5 (8.2%)	1 (5.3%)	4 (9.5%)	
no consent	5 (8.2%)	0 (0%)	5 (12%)	
**Do you work in a family-friendly environment? (yes, %)**	49 (78%)	15 (71%)	34 (81%)	0.5^2^
**Do you have the possibility for parental leave? (yes, %)**	25 (32%)	8 (35%)	17 (31%)	> 0.9^3^

^1^n (%)

^2^Fisher’s exact test

^3^Pearson’s Chi-squared test

### IR Career

**Fig. 2:**
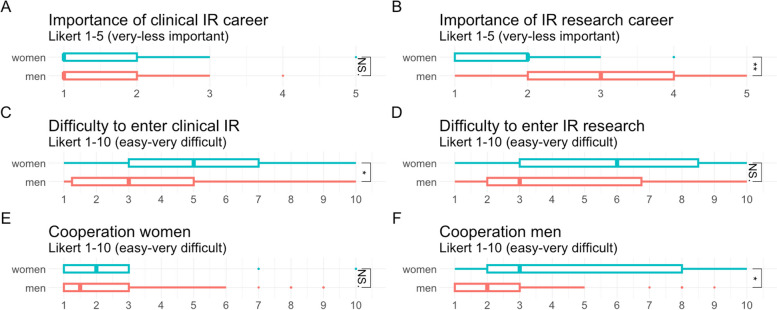
Clinical and academic IR career. **A**: Rating of the respondents about the importance of their clinical career. B: Rating of the respondents about the importance of their IR research career. **C**: Rating of the respondents about the difficulty to enter clinical IR. **D**: Rating of the respondents about the difficulty to enter IR research. E: Rating of the respondents about the cooperation with women. **F**: Rating of the respondents about the cooperation with men. Statistical significance markers: NS.- not significant, * *p* < 0.05; ** *p* < 0.01

All answers on the subset concerning IR career are displayed in Table 4. Both genders saw only limited changes regarding the role of women in IR in recent decades. According to all respondents, academic IR did not help to enter clinical IR. Only a small number of them had started with IR research to be able to enter clinical IR. Both genders were equally content with their career. Women and men were partially satisfied or less satisfied with the representation of women in IR and did not see differences in the representation over the last decades. Women considered their academic IR career more important than men (*p* = 0.044), while both, men and women rated the importance of their clinical work very high (Fig. 2A-B). Compared to men, women rated the entry into clinical IR as more difficult (*p* = 0.035). Both genders rated the start into IR research as challenging compared to clinical IR (Fig. 2C-D). Overall, the cooperation with other colleagues was rated as good. However, the female interventional radiologists reported lower ratings about the cooperation with men compared to the male interventional radiologists (*p* = 0.011) (Fig. 2E-F). When asked about the barriers to enter academic IR, the most respondents mentioned lack of (protected research) time and missing research infrastructure (Supplement 3).

**Table 4:** Summary of the questions about IR career, congress and committees

	Overall, *N* = 78^1^	women, *N* = 23^1^	men, *N* = 55^1^	*p*-value
**Was IR your initial aim? (yes, %)**	42 (62%)	17 (74%)	25 (56%)	0.2^2^
**Did you start research in IR to get access to clinical IR? (yes, %)**	13 (19%)	7 (30%)	6 (13%)	0.11^3^
**Did IR research help to access clinical IR? (yes, %)**	7 (54%)	3 (43%)	4 (67%)	0.6^3^
**How satisfied are you with your career so far?**				0.10^3^
satisfied	35 (56%)	8 (38%)	27 (66%)	
rather satisfied	20 (32%)	9 (43%)	11 (27%)	
partially satsified	6 (9.7%)	3 (14%)	3 (7.3%)	
less satisfied	1 (1.6%)	1 (4.8%)	0 (0%)	
**Have you noticed any differences in the representation of women in IR in the last decades?**				0.2^3^
no difference	19 (35%)	4 (25%)	15 (39%)	
to the negative	3 (5.6%)	2 (12%)	1 (2.6%)	
to the positive	32 (59%)	10 (62%)	22 (58%)	
**Are you satisfied with the representation of women in IR?**				0.2^3^
satisfied	4 (6.5%)	0 (0%)	4 (9.8%)	
rather satisfied	9 (15%)	1 (4.8%)	8 (20%)	
partially satsified	22 (35%)	8 (38%)	14 (34%)	
less satisfied	15 (24%)	6 (29%)	9 (22%)	
not satisfied	12 (19%)	6 (29%)	6 (15%)	
**What is the dominating gender in IR?**				> 0.9^3^
equally distributed	1 (1.4%)	0 (0%)	1 (2.2%)	
men	68 (99%)	23 (100%)	45 (98%)	
**Do you hold a position in committees? (yes, %)**	38 (49%)	11 (48%)	27 (49%)	> 0.9^2^
**Do you feel perceived at congresses? (yes, %)**	49 (80%)	10 (53%)	39 (93%)	< 0.001^3^
**Do you feel content at congresses? (yes, %)**	58 (89%)	15 (71%)	43 (98%)	0.004^3^
**Do you feel connected at congresses? (yes, %)**	42 (64%)	8 (36%)	34 (77%)	0.002^3^
**Do you actively network? (yes, %)**	45 (69%)	12 (60%)	33 (73%)	0.4^2^

^1^n (%)

^2^Pearson’s Chi-squared test

^3^Fisher’s exact test

### Funding, support and cooperation

**Fig. 3:**
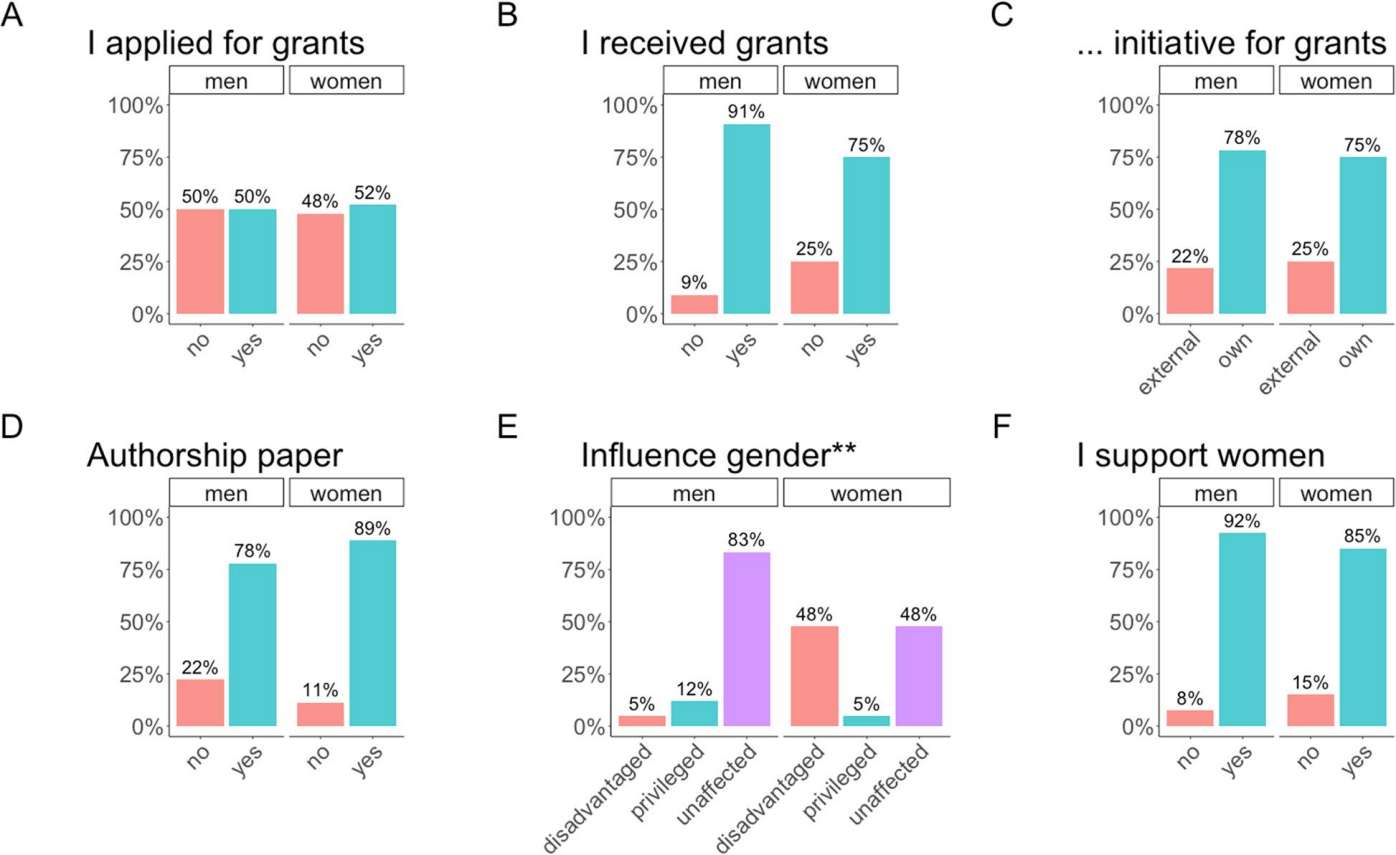
Bar graphs summarizing the questions about grants and support. **A** Proportion of women and men who applied for grants. **B** Proportion of women and men who received for grants. **C** Own or external initiative for the application for grants among women and men. **D** Proportion of women and men who had authorships on papers about topics where they made relevant contributions to. **E** Responses of women and men how their gender influenced their career. (F) Responses of women and men whether they support women. Statistical significance markers: NS.- not significant, * *p* < 0.05; ** *p* < 0.01

All answers about funding and support are summarized in Fig. 3. Half of the respondents had applied for grants and the majority had received grants (Fig. 3A-B). Amongst the applicants for grants and funding, 34% were women. Three fourths of both, men and women applied on their own initiative (Fig. 3C). One woman had received a grant especially for women. Three fourths of both, men and women indicated to be first or last author on papers about their own research topics (Fig. 3D). Women felt a 10-fold more often disadvantaged by their gender and men mostly felt disaffected (Fig. 3E, *p* < 0.001) in regard to their academic career. No difference in the support of women was found between men and women (Fig. 3F).

#### Congress and committees

Corresponding to the respondents, men dominate the national IR community. Overall, a large part of the respondents felt content and perceived at congresses. Table 4 demonstrates all comparisons for the situation at congresses and in committees. Comparable proportions of men and women reported that they were actively networking in the professional society, and that they held positions in committees. However, women felt less noticed at conferences, less confident, and less connected.

## Discussion

This study presents a status quo of academic IR in Germany. Key findings include the observations that academic IR benefited from protected research time. Protected research time correlated with grant funding. More female chief physicians granted protected research time. Fewer female faculty felt recognized at professional meetings.

Most of the IR researchers in our survey were younger than 46, especially the women were younger. These findings correspond with a review of gender demographic data of the Society of Interventional Radiology (SIR) showing that a doubling of female members was especially driven by trainee members [17].

Considering the low absolute numbers of the women there are only a few female senior researchers in IR. The higher mean age of men can be explained by the high rate of senior and chief physicians who took part at the survey. At the same time, it must be interpreted as low level of interest of younger men in getting surveyed. Of note, we observed a high response rate of women, with one third of the women compared to less than 10% men, implying a great interest of women in the survey’s topic. Compared to a survey among young interventional radiologists in Germany, the percentage of full-time employment was higher in our study (88% vs. 94%) [8], especially among men.

This survey documents comparable numbers of female chief physicians to previous studies [18]. 15% of the German university hospital chief physicians in radiology and 30% of the senior physicians in general are women [19]. This collective had less senior physicians, suggesting an overall gap in leading roles and missing role models in Germany.

A recent survey from the SIR focusing on mentoring in IR, reported that mentors gave significantly less guidance and direction to female medical students and residents in the field of IR education and finding a research mentor was a challenge for female residents [20]. Matsumoto et al. also found that female students often did not have a mentor of their gender [21]. These findings correspond with our observation that women had significant greater difficulties to enter clinical IR while the entry to IR research was rated equally. Especially in a scientific structure where IR research and direct patient care are closely linked, as it is in Germany, mentorship is of paramount importance. Data from this survey further suggest that women obviously try to enter IR via research activities. In a large survey, Goldman et al. found that compared to diagnostic radiology, the influence of a mentor had a significant positive impact on the decision to pursue a career in IR whereas the competitiveness had a negative impact [22]. Similar results were found by Xiang et al. in a large survey among IR trainees [23]. Thus, it is vital to set the fundaments for young researchers very early by providing mentorship, for example, through funding and mentorship programs.

According to this survey, female chief physicians provide the opportunity for protected research time significantly more often, resulting in an even higher percentage of respondents with protected research time than published in a current international survey among radiology trainees (60% vs. 38%) [24]. The issue of protected research time is of special interest in Germany, were no differentiation between clinical fellowship and research fellowship is made. Interventional radiologists who want to do research have to face up to both, clinical and research tasks, at one time. In other recent surveys among radiology trainees, the authors found that lack of time was a relevant barrier to research [11, 25]. Although our sample is small and only represents German faculty, we conclude that female chief physicians already recognized the importance of protected research time and are providing their employees with dedicated time for research activities. Of note, the success of this measure is evident in the survey results, as respondents who reported having access to such dedicated time demonstrated significantly higher rates of grant applications and more successful grant applications. Additionally, especially large institutions, i.e. university hospitals with their scientific focus, were able to realize protected research time. Many young medical graduates chose university hospitals to pursue a research career. Therefore, those large institutions play a key role when it comes to shaping a strong and diverse academic faculty in Germany. Future specific measures such as attracting more women to IR in general, protected research days for IR researchers and specific financial resources for IR research should pursue this goal giving possibility to foster more research activities and more leading female IR researchers.

In 2020, only 12% of the IR residency program directors in the United States were women [26]. This rate is lower than in general radiology, which nearly equals the average percentage of female program directors over all specialties with a bit over 25% [27]. Consequently, female program directors who serve as visible role models are missing in IR. This is of special importance as Long et al. showed a significant correlation of the percentage of female program directors with the percentage of female residents [27]. Work/ home balance and networking were rarely part of mentorship activities [20]. These topics could be fostered by female program directors having experienced similar barriers during their career. In keeping to previous findings, our respondents indicated that it is harder for women to manage family and work [18]. Taken together with the small number of role models, one of the future tasks for academic IR is to establish supportive networks and mechanisms of substantial support such as guidance for first and last authorships, invitations as speakers for podium and plenary sessions – not to fulfill a women’s quota but because of expertise – no, introduction into existing networks, and research time, especially for women. Recent data from the SIR shows that despite same professional qualification senior men dominated annual meetings [28]. It would be worthwhile to track demographic data of German authorships and congresses as well to objectify if there are similar patterns. This would be a strong commitment of leading interventional radiologists on gender equity in IR. Notably, this commitment is needed in the light of the German paradoxon with a large output in important IR journal articles but the lowest rate of women authorships in Europe [13]. Particularly for IR physicians with children special funding for childcare or childcare workers as well as more possibilities to work part-time is important to address the private challenges of managing both, private responsibilities and career.

According to our survey, three fourths of the women had the intention to pursue a career in IR from early on and more of them engaged in IR research with the aim of entering clinical IR. But, although not reaching significance, for more women IR research did not lead to a start in clinical IR. Hence, our data indicate that clinical IR might be a door opener for research activities in IR. All respondents rated their clinical career as very important. Differences, however, were found in the rating of the academic career. Knowing that this survey collected a subjective feeling and not a quantifiable value it seems that women put high pressure on themselves. There should be no demand that all women in IR enter leadership. Voytko et al. conducted a longitudinal survey on participants of a mentoring program and reported a high relevance of mentors to determine career goals, provide constructive feedback, give personal support and being a role model. According to their data, all mentees benefited from the relationship to the mentor [29]. Inadequate mentorship was one of the most important factors that limiting research time in a large Canadian survey among residents in general radiology [25]. Interestingly, the differences in the ratings to enter clinical career might also explain the high importance of academic career as it was harder for the respondents to enter IR at the very beginning, namely clinical IR. As mentioned above, the ratings only represent subjective feelings and it is unclear whether the ratings reflect the real circumstances.

When it comes to collaboration, strong networks are the key to success. Like other studies, our respondents unanimously confirmed that the dominating gender in IR is male [18]. At the same time women rated the cooperation with their male colleagues as more difficult. A retrospective long-term analysis about gender trends in radiology authorship reported a significant tendency of physicians to publish with physicians of the same gender [30]. This is an obstacle in the career of female IR researchers. Previous publications on collaboration metrics among researchers showed an association of female first or last authorships with more contributing departments and institutions implicating that distinctive network take shape in those publications [31]. Taken together with a positive trend of women authorships in the last decade [32], this particular feature could serve as a chance in IR research. All our respondents supported women bearing high potential to support each other and perhaps ensure more diversity. Our female respondents were surrounded by significantly more female interventional radiologists, which might demonstrate focal manifestations of female networks.

Although not reaching significance, men were 16% more successful with their applications for grants than women. This finding stands in contrast to the application rates that are equal between both genders, and is a known phenomenon in science. Wittman et al. analyzed the success of a large number of applications in all investigator-initiated grant programs of the Canadian Institutes of Health Research over a five-year period. They found that success rates for female applicants in the foundation program that focused on the scientist were significantly lower than those for male applicants. At the same time, evaluations that focused on the proposed science did not yield different success rates for men and women. These data suggest a gender gap in evaluations, particularly for female applicants, regardless of the quality of their proposal [33]. Similar results have been reported in large studies in the Netherlands and the USA [34, 35]. Corresponding studies in the field of IR do not currently exist. Therefore, it is unclear whether the differences in success of the proposals in IR are due to gender bias or different quality of applications. Further research should address the issue of gender-specific success rates to evaluate the assessment procedures for funding and grants in IR in Germany.

Women felt less noticed and less connected at congresses. Women felt 10 times more disadvantaged by their gender. Compared to data from a large world-wide survey about research activities in radiology among residents this is double the amount, suggesting a special gender-specific barrier in IR. At the same time, half as many men saw themselves disadvantaged by their gender in our survey [11]. Equity in IR research encompasses the same possibilities to participate in research programs, to receive funding, and to have grant applications scored without bias. Important is the commitment of senior leaders in German IR to implement these fundamental changes. This survey is a first step towards cultural changes in IR research in Germany. This change is complemented by a slowly growing number of female DEGIR members (2021:14%, 2023:17%).

This study has some limitations. This was a voluntary, not validated survey resulting in a possible response bias. Unfortunately, the few non-binary respondents had to be excluded from analytic statistics due to privacy reasons. It would be interesting, and it is important, to explore their situation more in depth. The cohort size was 78 respondents who were involved in IR research and not all questions were obligatory. Hence, some questions were only answered by a small group of respondents. Thus, we interpret the results with caution. Additionally, the high response rate of women having more imponderabilities might skew the data towards a more distressed view and not reflect the real status of women or young interventional radiologists. Still, the cohort seems to be representative when comparing the demographics with existing studies in IR and bearing the high absolute number of responses by men and the high response rate of women in mind. Further studies with more participants are needed for closer studies on subgroups to find out if differences attributed to gender might also be attributable to age or family status.

## Conclusion

In conclusion, women and men did research under the same circumstances in terms of family-friendliness of their institutions and investment of time during unpaid working hours in research. However, women were underrepresented in IR research. Protected research time was granted by more women. Especially protected research time without additional obligations in the clinical routine should be a focus of future mentoring programs as it is correlated with grant funding. Currently many women in IR research are younger and not in leading positions. Female mentors are needed to further advance academic IR in Germany.

### Supplementary Information

Supplementary Material 1

Supplementary Material 2

Supplementary Material 3

Supplementary Material 4

Supplementary Material 5

## Data Availability

The datasets used and/or analysed during the current study are available from the corresponding author on reasonable request.

## Abbreviations

DeGIR: The German Society for Interventional Radiology and Minimally Invasive Therapy
IR: Interventional Radiology
SIR: Society of Interventional Radiology
