# Supplementary material for: The status of academic interventional radiologists in Germany with focus on gender disparity: how can we do better?
Source: CVIR Endovasc. 2024 May 16;7:47. doi: 10.1186/s42155-024-00456-4 (PMC11098981; doi:10.1186/s42155-024-00456-4)
Supplement: Supplementary file 1 — Supplementary Material 1 [file 42155_2024_456_MOESM1_ESM.pptx]

## Slide 1
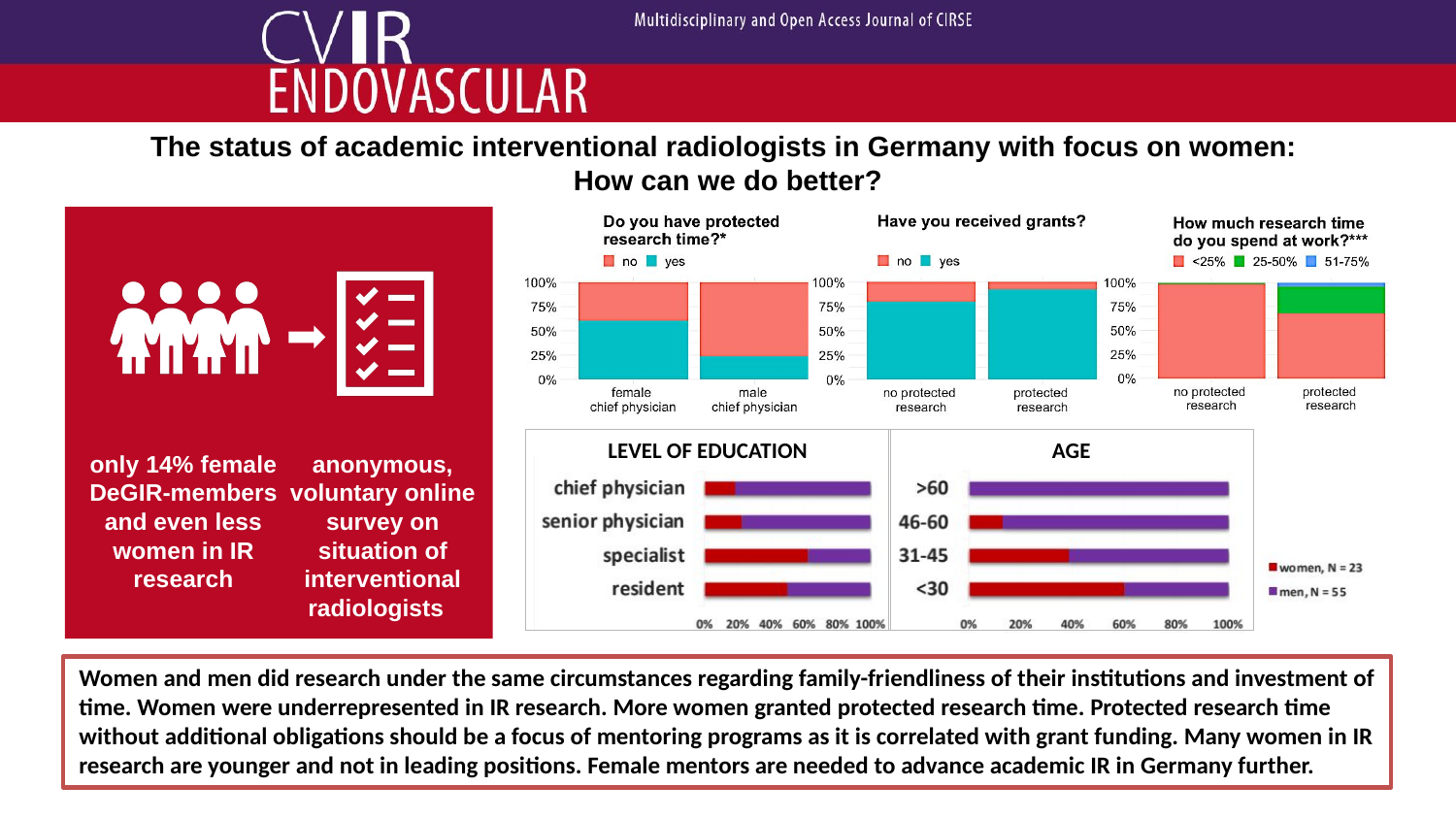

# The status of academic interventional radiologists in Germany with focus on women: How can we do better?
only 14% female DeGIR-members and even less women in IR research
anonymous, voluntary online survey on situation of interventional radiologists
LEVEL OF EDUCATION
AGE
Women and men did research under the same circumstances regarding family-friendliness of their institutions and investment of time. Women were underrepresented in IR research. More women granted protected research time. Protected research time without additional obligations should be a focus of mentoring programs as it is correlated with grant funding. Many women in IR research are younger and not in leading positions. Female mentors are needed to advance academic IR in Germany further.
