# Supplementary material for: The status of academic interventional radiologists in Germany with focus on gender disparity: how can we do better?
Source: CVIR Endovasc. 2024 May 16;7:47. doi: 10.1186/s42155-024-00456-4 (PMC11098981; doi:10.1186/s42155-024-00456-4)

Supplement 2

49 responders answered the open-ended question ‘What makes it harder for women to manage career and family?’. The content of the answers was clustered in topics. Finally, the frequency of the occurence of each topic was counted and ranked in descending order. Below the list, a word cloud of all answers is presented.

Topic (frequency)

1. traditional role model with more family obligations for women (16)
2. more downtime during pregnancy or sick-leave for a child (14)
3. not enough time (7)
4. pregnancy (7)
5. others (5)
6. not enough childcare (5)
7. insufficient working time model (4)
8. (on-call) duty (2)


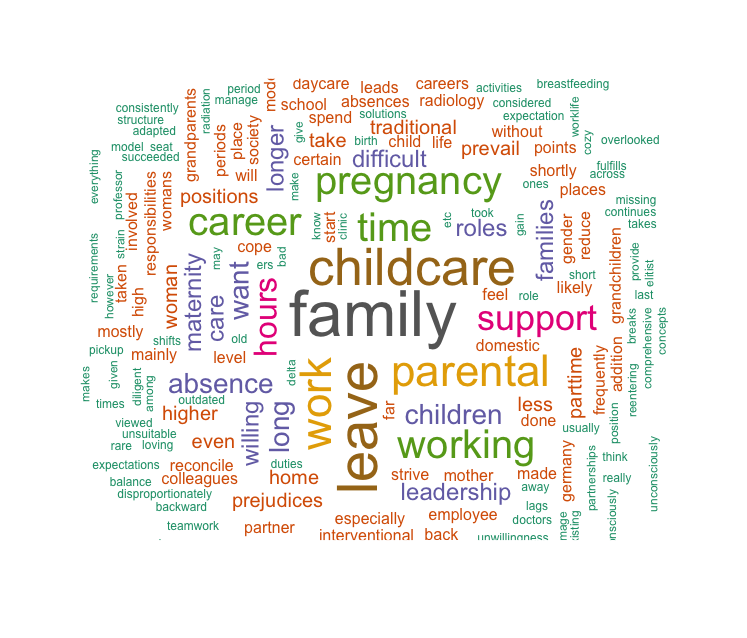

Supplement: Supplementary file 2 — Supplementary Material 2 [file 42155_2024_456_MOESM2_ESM.docx]
