# Supplementary material for: The status of academic interventional radiologists in Germany with focus on gender disparity: how can we do better?
Source: CVIR Endovasc. 2024 May 16;7:47. doi: 10.1186/s42155-024-00456-4 (PMC11098981; doi:10.1186/s42155-024-00456-4)

Supplement 3

32 responders answered the open-ended question ‘What were the barriers to enter academic IR?’. The content of the answers was clustered in topics. Finally, the frequency of the occurence of each topic was counted and ranked in descending order. Below the list, a word cloud of all answers is presented.

Topic (frequency)

1. No time or protected research time (16)
2. No research infrastructure (9)
3. missing know-how (5)
4. missing support/mentoring (4)
5. preference of male colleagues (2)
6. no problems (1)


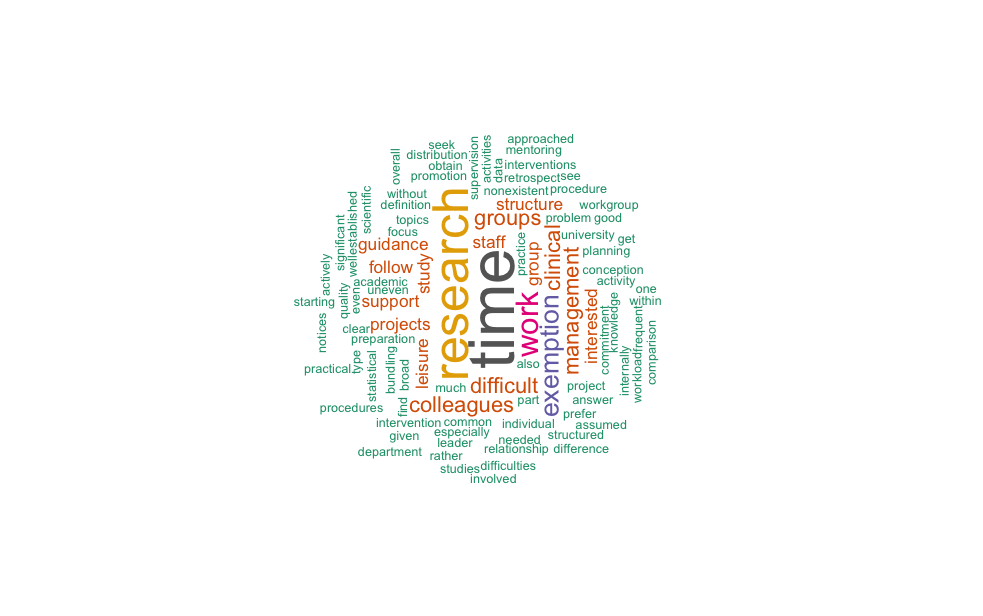

Supplement: Supplementary file 3 — Supplementary Material 3 [file 42155_2024_456_MOESM3_ESM.docx]
