# Supplementary material for: The status of academic interventional radiologists in Germany with focus on gender disparity: how can we do better?
Source: CVIR Endovasc. 2024 May 16;7:47. doi: 10.1186/s42155-024-00456-4 (PMC11098981; doi:10.1186/s42155-024-00456-4)
Supplement: Supplementary file 4 — Supplementary Material 4 [file 42155_2024_456_MOESM4_ESM.docx]

# Supplement

| Item |
| --- |
| **age** |
| <30 |
| 31-45 |
| 46-60 |
| >60 |
| **level of education** |
| resident |
| specialist |
| senior physician |
| head of department |
| **hospital type** |
| other hospital |
| teaching hospital |
| university hospital |
| **number of beds** |
| 50-199 |
| 200-399 |
| 400-799 |
| >800 |
| **income** |
| equal |
| main earner |
| side earner |
| **employment status** |
| full-time |
| part-time |
| **What is the gender of your head?** |
| diverse |
| female |
| male |
| **What is the gender of your supervisor?** |
| diverse |
| female |
| male |
| **What is the gender of your researchgroup leader?** |
| diverse |
| female |
| male |
| **What is the number of your colleagues?** |
| Total number |
| **How many interventional radiologists work at your department?** |
| Total number |
| **How many female interventional radiologists work at your department?** |
| Total number |
| **How much of your working time do spend with interventions?** |
| <25% |
| 25-50% |
| 51-75% |
| >75% |
| **Do you get time to do research?** |
| Yes |
| No |
| **How much research time do spend at work?** |
| <25% |
| 25-50% |
| 51-75% |
| >75% |
| **How much research time do you spend in your free time?** |
| <25% |
| 25-50% |
| 51-75% |
| >75% |
| **family status** |
| partnership |
| single |
| **Do you have children?** |
| Yes |
| No |
| **number of children** |
| Total number |
| **Who is responsible for the children?** |
| equal |
| no |
| yes |
| **Do you have the possibility for daycare?** |
| Yes |
| No |
| **Are you a single parent?** |
| Yes |
| No |
| **For women it is harder to manage children and career. *** |
| consent |
| rather consent |
| partial consent |
| less consent |
| no consent |
| **Do you work in a family-friendly environment?** |
| Yes |
| No |
| **Was IR your initial aim?** |
| Yes |
| No |
| **Did you start research in IR to get access to clinical IR?** |
| Yes |
| No |
| **Did IR research help to access clinical IR?** |
| Yes |
| No |
| **How satisfied are you with your career so far?** |
| satisfied |
| rather satisfied |
| partially satsified |
| less satisfied |
| Not satisfied |
| **How important is your clinical career?** |
| Likert scale 1-5 with 1 being important |
| **What was the level of difficulty to enter clinical IR?** |
| Likert scale 1-10 with 1 being very easy |
| **How important is your academic career?** |
| Likert scale 1-5 with 1 being important |
| **What was the level of difficulty to enter academic IR? *** |
| Likert scale 1-10 with 1 being very easy |
| **How is the cooperation with male colleagues?** |
| Likert scale 1-10 with 1 being very good |
| **How is the cooperation with female colleagues?** |
| Likert scale 1-10 with 1 being very good |
| **Have you noticed any differences in the representation of women in IR in the last decades?** |
| no difference |
| to the negative |
| to the positive |
| **Are you satisfied with the representation of women in IR?** |
| satisfied |
| rather satisfied |
| partially satsified |
| less satisfied |
| Not satisfied |
| **What is the dominating gender in IR?** |
| equally distributed |
| women |
| men |
| **Do you hold a position in committees?** |
| Yes |
| No |
| **Do you feel perceived at congresses?** |
| Yes |
| No |
| **Do you feel content at congresses?** |
| Yes |
| No |
| **Do you feel connected at congresses?** |
| Yes |
| No |
| **Do you actively network?** |
| Yes |
| No |

* This question was followed by the possibility of a free-text answer.
